# Anæsthetics

**Published:** 1866-01

**Authors:** 


### ANÆSTHETICS.

An anonymous writer, in a communication to the *Boston Medical and Surgical Journal* (September 7), asserts that the safety of *inhalations of nitrous oxide* for producing anæsthesia, and objecting seriously to its indiscriminate use by ignorant and unskilled (dental?) practitioners, urges a trial of it by professional men.

"When properly prepared and properly administered, we cannot but regard it among the safest of inhalants. That prepared by ourselves we have inhaled to entire anæsthesia *one hundred and ten* times during the past ten months, with a very perceptible improvement in our general health, while we have given this gas to others for sanitary and anæsthetic objects some three hundred times in the same period; and in no single instance do we remember meeting with the least unfavorable result. In certain dental establishments in this city it is almost, if not entirely, used for anæsthesia. The proprietor of one of these recently stated to us that it had been employed by him for anæsthesia in nearly eight thousand cases, with not a serious result.

But by some it is urged that, while perhaps safe for brief anæsthesia, this cannot be predicated of the gas in protracted operations. In at least three instances, we have known entire anæsthesia maintained during the surgical operations lasting from four to seven minutes, with the most happy effects on awakening. Others assure us of success with this in cases of anæsthesia much more protracted. From what we have heard and witnessed ourselves, we have reason to believe that entire insensibility to pain may be safely maintained for a longer period than with ether or chloroform. This may be inferred from the vitalizing character of nitrous oxide, which maintains the temperature and pulse by oxydizing the blood, while it is well known that the effects of ether, or more particularly chloroform, are the reverse of this.

To be safe, nitrous oxide, like chloroform, should be properly made and administered. In making this gas, we employ an automatic regulator of the heat, confining the nitrate of ammonia in the flask to a temperature scarcely above 400 degrees. The results of decomposition at this point are quite different from those where the heat of the salt is fluctuating, rising at times to 500 degrees, and even to 600 degrees, as is common by the ordinary process of heating. Thus prepared, the gas in its passage to the gas-holder is forced through five one-and-a-half gallon glass washers, filled with an alkaline solution and pure water ; each washer being provided with perforated glass tubes for finely dividing and washing the gas. Thus five distinct plunges are made through as many feet of water, when the gas is passed into a zinc receiver, where it is confined over water. This whole process is simple, safe and economical.

For inhaling we employ an improved valved apparatus, drawing the gas directly from the zinc holder and exhaling into the open air. With this arrangement perfect anæsthesia in its most agreeable form may be produced, while even the most sensitive suffer not the least inconvenience."

Dr. Nunneley, of Leeds, brings forward two new anæsthetics as substitutes for chloroform, the *bromide of ethyl* and the *chloride of olefiant gas*, each of which he believes to possess important advantages over chloroform. They both act speedily, pleasantly and well. The patient might be kept insensible for any length of time, while the most painful and prolonged operations were being performed. No disagreeable symptoms had, in any case, resulted from their use. The original difficulty and cost of their manufacture has been overcome, and, should their use become general, they can be made at a cost not exceeding that of chloroform, if not at less.—*Amer. Jour. Med. Science, Oct., from Medical Times and Gazette.*

